# Complete genome of *Bacillus anthracis* strain ter21 from an infected zoo pony in Ternopil, Ukraine, 2021

**DOI:** 10.1128/mra.01301-25

**Published:** 2026-06-22

**Authors:** Vitaliy Bolotin, Jeremy Buttler, Oksana Kravtsova, Oleksandr Pishchanskyi, Vitaliy Ukhovskiy, Galina Aliekseieva, Vasyl Arefiev, Ganna Kovalenko, Anton Gerilovych, Eric Bortz

**Affiliations:** 1 State Scientific and Control Institute of Biotechnology and Strains of Microorganisms (SSCIBSM), Kyiv, Ukraine; 2 National Scientific Center Institute of Experimental and Clinical Veterinary Medicine (NSC IECVM), Kharkiv, Ukraine; 3 State Scientific and Research Institute of Laboratory Diagnostics and Veterinary and Sanitary Expertise (SSRILDVSE), Kyiv, Ukraine; 4 Department of Biological Sciences, University of Alaska Anchorage 3291, Anchorage, Alaska, USA; 5 Department of Population Health and Disease Prevention, Joe C. Wen School of Population and Public Health, University of California 8788, Irvine, California, USA; 6 Institute for Problems of Cryobiology and Cryomedicine of the National Academy of Sciences of Ukraine 309031, Kharkiv, Ukraine; 7 PSI One Health Scientific and Research Institute, Kharkiv, Ukraine; 8 Pathogenomics Lab, Department of Computer Science, Kyiv School of Economics, Kyiv, Ukraine; Wellesley College, Wellesley, Massachusetts, USA

**Keywords:** *Bacillus anthracis*, anthrax, nanopore, DNA sequencing, genome, Ukraine, horse, zoo

## Abstract

Using rapid nanopore whole-genome sequencing, we assembled the genome of *Bacillus anthracis* strain ter21 (5,229,480 bp), a Tsiankovskii-I group isolate cultured from a fatal case in 2021 of a pony from a zoo in Ternopil, Ukraine, identifying virulence plasmid pXO1 that encodes anthrax toxin, and pXO2.

## ANNOUNCEMENT

In recent years (2007–2022), Ukraine has suffered sporadic anthrax outbreaks, with at least 15 fatal cases in domesticated animals (cattle, goats, pigs, dogs, and horses), with zoonotic spillover to eight human cases of cutaneous anthrax (1). In September 2021, a suspected case of anthrax was reported in a pony (*Equus ferus caballus*) that had died suddenly at a zoo in Ternopil, Ukraine (2). Upon necropsy, gross pathology revealed classical signs of systemic anthrax: pneumonia, gastroenteritis, lymphadenitis, and splenomegaly. Presumptive diagnosis of *Bacillus anthracis* infection was confirmed by positive gram stain, motility, and penicillin sensitivity test (“pearl necklace” observed) from bacteriological culture of the organism from pathological samples (internal organs) on sheep blood agar, incubated at 37°C for 24 h. In an effort to genetically characterize this especially dangerous zoonotic pathogen reservoirs in Ukraine (3), we sequenced the genome of *B. anthracis* strain from this outbreak.

Bacteria from a single colony culture archived at −70°C in CryoInstant (VWR, USA) were used directly for DNA isolation with a HighPure PCR Template Preparation Kit (Roche Diagnostics, USA) conducted under enhanced Biosafety Level 3 (BSL3) in a secure laboratory in Kyiv, Ukraine (SSCIBSM, under approved protocol No. 7, 30 August 2024). Molecular sequencing of purified bacterial DNA (1.4 µg) used Oxford Nanopore Technologies (ONT) RBK114.24 V14 chemistry, Rapid Barcoding Library protocol, on an ONT MinION Mk1B device and FLOMIN114 flow cell (R10, V10.4). High-accuracy (HAC) basecalling (in MinKNOW v.24.06.5; model r10.4.1_e8.2_400bps_hac@v4.3.0; min. quality, *Q* = 9; mean *Q* = 15) included read filtering (ONT Guppy v.6) (3). Sequence reads (252,803 reads; *N*_50_ = 2,471 nt; mean/median *Q* = 15) were assembled (three contigs) into one bacterial chromosome contig (5,229,480 bp; mean read depth 48.5×; GC content 35.38%) by *de novo* assembly (Flye v.2.9.4), rotated and checked for completeness by reference-based hybrid assembly (Raven v.1.8.3 and minimap2 v. 2.28-r1209) to *B. anthracis* strain Ames Ancestor reference core genome (AE017334.2), and annotated in NCBI PGAP (GeneMarkS-2+v.6.10). Extrachromosomal virulence plasmids (two contigs) assembled as pXO1 (181,759 bp; mean depth 142.1×; GC 32.53%; reference sequence NZ_AP018444), encoding anthrax lethal factor (LF), protective antigen (PA), and edema factor (EF) genes; and pXO2 (94,711 bp; mean depth 116.5×; GC 33.05%; reference sequence NC_007323) encoding capsule capB/C genes. PA and LF associate to generate lethal toxin (LT), and PA and EF generate edema toxin (ET), key virulence toxins in anthrax infection that are defining for *B. anthracis* among *Bacillus* spp. (4).

**Fig 1:**
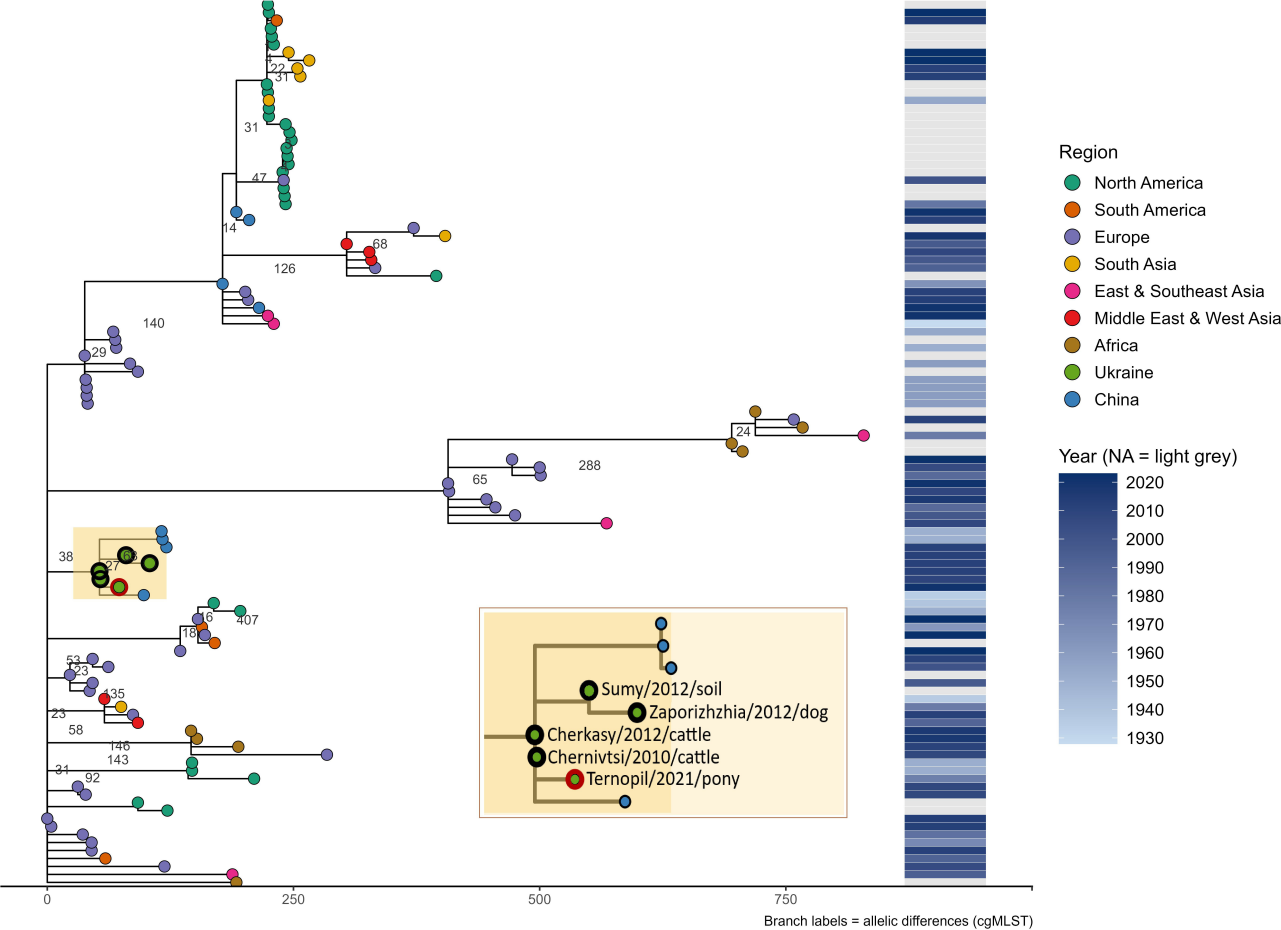
Minimum spanning tree (MST) of *Bacillus anthracis* isolates based on cgMLST allelic distances. Each node represents an isolate (*n* = 108 isolates), colored by region of origin. Branch labels indicate the number of allelic differences between connected nodes. The heat strip on the right shows the year of isolation (darker shades = more recent). The highlighted clade includes Ukrainian isolates (outlined in black) from Chernivtsi (2010, cattle), Cherkasy (2012, cattle), Sumy (2012, soil), and Zaporizhzhia (2012, dog). The Ternopil (2021, pony) isolate from this study is outlined in red.

To understand the nature of this isolate, we analyzed the 2021 *B. anthracis* ter21 strain using VNTR32 database (MLVA bank v4_1, at: https://microbesgenotyping.i2bc.paris-saclay.fr/databases/view/9/), with the Ames Ancestor (AE017334.2) reference under default parameters (5). The closest VNTR32 strain to ter21 was BA-D12-MEL, isolated from an infected dog in 2012 in Zaporizhzhia, Ukraine (6). Core genome *B. anthracis* cgMLST scheme from Ridom (v.1.0, https://www.cgmlst.org/ncs/schema/Banthracis/) was used for comparative cgMLST analysis, with 3,900 of 3,803 loci detected (7). Along with historical strains of anthrax bacteria from across Europe, including Ukraine, we built a minimum spanning tree (GrapeTree v.1.5.0, visualized in ggtree v.3.16.0 in R) to identify the ter21 strain’s most closely related genetic neighbors (Fig. 1). Although sparse, the tree shows closest genetic relationships of *B. anthracis* ter21 and classifies it as a contemporary Eurasian A strain within the Eurasian Tsiankovskii-I group (A.Br.105) (Fig. 1). Antimicrobial resistance (AMR) genes typical in *Bacillus* spp. were predicted (AMRFinderPlus v.3.10; built-in BV-BRC v.3.54.6; https://github.com/BV-BRC), for example, beta-lactam AMR encoded by Bla1 and BcII core genes (8).

## Data Availability

Genomic data for this isolate are available in NCBI GenBank under BioProject number PRJNA1308119, BioSample number SAMN50683810, NCBI accession numbers CM148503.1, JBSCFG010000001.1, JBSCFG010000002.1, and JBSCFG010000003.1, and raw data are available in the Sequence Read Archive (SRA) under accession number SRX30152983. Detailed descriptions of bioinformatics tools and consensus sequences are also available at: https://github.com/jeremyButtler/2024-UKR-Banthracis (under commit hash: https://github.com/jeremyButtler/2024-UKR-Banthracis/commit/645b1857cca6d4817179f9e3e391f764750e4a5b).
